# Supplementary material for: In Silico Prediction of Metabolic Reaction Catalyzed by Human Aldehyde Oxidase
Source: Metabolites. 2023 Mar 19;13(3):449. doi: 10.3390/metabo13030449 (PMC10059660; doi:10.3390/metabo13030449)
Supplement: Supplementary file 1 [file metabolites-13-00449-s001.zip › metabolites-2276878-supplementary.pdf]

Supporting Information

In Silico Prediction of Metabolic Reaction Catalyzed by Human Aldehyde Oxidase

Mengting Huang, Keyun Zhu, Yimeng Wang, Chaofeng Lou, Huimin Sun, Weihua Li, Yun Tang * and Guixia Liu *

Shanghai Frontiers Science Center of Optogenetic Techniques for Cell Metabolism,

Shanghai Key Laboratory of New Drug Design, School of Pharmacy,

East China University of Science and Technology, Shanghai 200237, China

* Correspondence: ytang234@ecust.edu.cn (Y.T.); gxliu@ecust.edu.cn (G.L.);

Tel.: +86-21-6425-0811 (G.L.)

Table Legends

Table S1. The substrates information collected from references

Table S2. The number of the substrate and the SOM associated with hAOX in the training set and external test set

Table S3. The parameters and value of feature selection methods

Table S4. Parameters of the machine learning methods

Table S5. The definition of atom features for the WLN model

Table S6. The definition of bond features for the WLN model

Table S7. Configuration for the WLN model

Table S8. Configuration for the Transformer model

Table S9. The predicted results of the transformer-baseline model on the test set

Table S10. The predicted results of the transformer-transfer learning model on the test set

Figure Legends

Figure S1. Examples of hAOX -catalyzed reactions.

Figure S2. The performance of the best fingerprint-based model on training set, 10-fold and test set.

Figure S3. Some examples of results predicted by the three different models.

Table S1. The substrates information collected from references

No.	Name	Reference	React	Product
1	RO1	[6]	CC(CO)NC1=NC=C2C(N(CC(O)C)C(C(OC3=CC=C(F)C=C3F)=C2)=O)=N1	CC(NC1=NC(O)=C2C(N(C(C(OC3=CC=C(C=C3F)F)=C2)=O)CC(C)O)=N1)CO
2	SGX523	[47]	CN1N=CC(C(C=C2)=NN3C2=NN=C3SC4=CC=C(C=CC=N5)C5=C4)=C1	CN1N=CC(C(C=C2)=NN3C2=NN=C3SC4=CC=C5C=CC(O)=NC5=C4)=C1
3	RS-8359	[48]	CC1=C(C#N)C=CC(NC2=NC=NC3=C2CCC3O)=C1	CC1=C(C=CC(NC2=NC(O)=NC3=C2CCC3O)=C1)C#N
4	XK-469	[48]	CC(C(O)=O)OC1=CC=C(OC2=NC(C=C(Cl)C=C3)=C3N=C2)C=C1	CC(OC1=CC=C(C=C1)OC2=NC3=C(N=C2O)C=CC(Cl)=C3)C(O)=O
5	2-Aminopurine	[9]	NC1=NC=C2C(NC=N2)=N1	NC1=NC=C2C(NC(O)=N2)=N1
6	2-Hydroxypurine	[49]	O=C1N=CC2=C(NC=N2)N1	O=C1N=C(O)C2=C(N1)NC=N2
7	2-Mercaptopurine	[49]	S=C1NC2=C(N=CN2)C=N1	S=C1NC2=C(C(O)=N1)N=CN2
8	4-Hydroxypteridine	[49]	O=C1C2=C(N=CC=N2)NC=N1	O=C1C2=C(NC(O)=N1)N=CC=N2
9	4-Methylacridine	[49]	CC1=CC=CC2=CC3=CC=CC=C3N=C21	CC1=CC=CC2=C(O)C3=CC=CC=C3N=C21
10	6-Mercaptopurine	[50]	S=C1NC=NC2=C1N=CN2	S=C1NC=NC2=C1N=C(O)N2
11	6-Methylpurine	[51]	CC1=C2C(NC=N2)=NC=N1	CC1=C2C(NC(O)=N2)=NC=N1
12	Adenine	[49]	NC1=NC=NC2=C1N=CN2	NC1=NC=NC2=C1N=C(O)N2
13	Carbazeran	[52]	COC1=CC(C(N2CCC(OC(NC)=O)CC2)=NN=C3)=C3C=C1OC	COC1=CC2=C(C=C1OC)C(O)=NN=C2N3CCC(CC3)OC(NC)=O
14	Cinnoline	[53]	C1(C=CC=C2)=C2N=NC=C1	OC1=CN=NC2=C1C=CC=C2
15	Hypoxanthine	[9]	O=C1NC=NC2=C1NC=N2	O=C1NC=NC2=C1NC(O)=N2
16	Methotrexate	[54]	NC1=C2C(N=CC(CN(C)C3=CC=C(C(NC(CCC(O)=O)C(O)=O)=O)C=C3)=N2)=NC(N)=N1	NC1=C2C(N=C(O)C(CN(C3=CC=C(C=C3)C(NC(C(O)=O)CCC(O)=O)=O)C)=N2)=NC(N)=N1
17	O-Benzyloxyguanine	[52]	NC1=NC(N=CN2)=C2C(OCC3=CC=CC=C3)=N1	NC1=NC2=C(C(OCC3=CC=CC=C3)=N1)NC(O)=N2
18	Phthalazine	[55]	C12=CC=CC=C1C=NN=C2	OC1=NN=CC2=CC=CC=C21
19	Zebularine	[56]	O=C1N=CC=CN1C2OC(CO)C(O)C2O	O=C1N=C(O)C=CN1C2OC(C(C2O)O)CO
20	Zoniporide	[48]	O=C(NC(N)=N)C(C=N1)=C(C2CC2)N1C3=CC=CC4=C3C=CC=N4	O=C(C(C=NN1C2=CC=CC3=C2C=CC(O)=N3)=C1C4CC4)NC(N)=N
21	Compound 1	[57]	CC1=C(C(NC(N)=N)=O)C=NN1C2=C3C(NC=N3)=CC=C2	CC1=C(C=NN1C2=C3C(NC(O)=N3)=CC=C2)C(NC(N)=N)=O
22	Imatinib	[58]	CN(CC1)CCN1CC2=CC=C(C(NC3=CC(NC4=NC(C5=CN=CC=C5)=CC=N4)=C(C)C=C3)=O)C=C2	CN1CCN(CC2=CC=C(C=C2)C(NC3=CC(NC4=NC(C5=CN=C(O)C=C5)=CC=N4)=C(C=C3)C)=O)CC1
23	SB-277011	[59]	N#CC1=CC(CCN(CCC2CCC(NC(C3=CC=NC4=C3C=CC=C4)=O)CC2)C5)=	N#CC1=CC2=C(C=C1)CN(CCC3CCC(CC3)NC(C4=CC(O)=NC5=C4C=CC=C

			C5C=C1	5)=O)CC2
24	Compound 2	[60]	CN(N=C1)C=C1C2=CN=C(N=NN3C(C(C=C4)=CN5C4=NC=C5)C)C3=N2	CN1N=CC(C2=C(O)N=C(C3=N2)N=NN3C(C)C(C=C4)=CN5C4=NC=C5)=C1
25	A-77-01	[58]	CC1=CC=CC(C2=NNC=C2C3=CC=NC4=CC=CC=C43)=N1	CC1=CC=CC(C2=NNC=C2C3=CC(O)=NC4=CC=CC=C43)=N1
26	AMG-900	[58]	NC1=NC=CC(C2=C(OC3=CC=C(NC4=NN=C(C5=CC(C)=CS5)C6=C4C=CC=C6)C=C3)N=CC=C2)=N1	OC1=CC(C2=C(N=CC=C2)OC3=CC=C(C=C3)NC4=NN=C(C5=C4C=CC=C5)C6=CC(C)=CS6)=NC(N)=N1
27	Bafetinib	[58]	CC1=CC=C(NC(C2=CC=C(CN3CC[C@H](N(C)C)C3)C(C(F)(F)F)=C2)=O)C=C1NC4=NC=CC(C5=CN=CN=C5)=N4	CC1=CC=C(C=C1NC2=NC=CC(C3=CN=C(O)N=C3)=N2)NC(C4=CC=C(C(C(F)(F)F)=C4)CN5CC[C@@H](C5)N(C)C)=O
28	CL-387785	[58]	BrC1=CC=CC(NC2=NC=NC3=CC=C(NC(C#CC)=O)C=C32)=C1	BrC1=CC=CC(NC2=NC(O)=NC3=CC=C(C=C32)NC(C#CC)=O)=C1
29	Lapatinib	[58]	O=S(CCNCC1=CC=C(C2=CC3C(N=CN=C3NC4=CC=C(C(Cl)=C4)OCC5=CC=CC(F)=C5)C=C2)O1)(C)=O	O=S(CCNCC1=CC=C(C2=CC3C(N=C(O)N=C3NC4=CC=C(C(Cl)=C4)OCC5=CC=CC(F)=C5)C=C2)O1)(C)=O
30	Lapatinib-M1	[58]	ClC1=CC(NC2=C3C(C=CC(C4=CC=C(CNCCS(=O)(C)=O)O4)=C3)=NC=N2)=CC=C1O	ClC1=CC(NC2=C3C(C=CC(C4=CC=C(O4)CNCCS(=O)(C)=O)=C3)=NC(O)=N2)=CC=C1O
31	LDN-193189	[58]	N1(C2=CC=C(C(C(C=N3)=CN4C3=C(C5=CC=NC6=CC=CC=C56)C=N4)C=C2)CCNCC1	OC1=NC2=CC=CC=C2C(C(C=N3)=C4N3C=C(C=N4)C(C=C5)=CC=C5N6CCNCC6)=C1
32	ML-347	[58]	COC1=CC=C(C(C=N2)=CN3C2=C(C4=CC=NC5=C4C=CC=C5)C=N3)C=C1	COC1=CC=C(C=C1)C(C=N2)=CN3C2=C(C=N3)C4=CC(O)=NC5=C4C=CC=C5
33	SB-525334	[58]	CC1=NC(C2=C(C3=CC(N=CC=N4)=C4C=C3)N=C(C(C)(C)C)N2)=CC=C1	CC1=NC(C2=C(N=C(N2)C(C)(C)C)C3=CC4=C(C=C3)N=C(O)C=N4)=CC=C1
34	Duvelisib	[58]	ClC1=C2C(C=C([C@H](C)NC3=NC=NC4=C3N=CN4)N(C5=CC=CC=C5)C2=O)=CC=C1	ClC1=C2C(C=C(N(C2=O)C3=CC=CC=C3)[C@@H](NC4=NC(O)=NC5=C4N=CN5)C)=CC=C1
35	Lu AF09535	[61]	O=C(C1=CN=CC(C)=N1)N[C@]23C[C@@H](C[C@H]4C3)C[C@](C4)(NC(C5=CC=NC(C)=N5)=O)C2	O=C(N[C@@]12C[C@H](C[C@](NC(C3=CC(O)=NC(C)=N3)=O)(C2)C4)C[C@H]4C1)C5=CN=CC(C)=N5
36	O6-benzylguanine	[52]	NC1=NC2=C(N=CN2)C(COC3=CC=CC=C3)=N1	NC1=NC2=C(N=C(O)N2)C(COC3=CC=CC=C3)=N1
37	LY3202626	[62]	FC1=CN=C(N2CC(N=C(N)SC3)(C4=CC(NC(C5=NC=CN=C5)=O)=CC=C4F)C3C2)N=C1	FC1=CN=C(N=C1)N2CC3(C4=CC(NC(C5=NC=C(N=C5)O)=O)=CC=C4F)N=C(SCC3C2)N
38	VX-509	[63]	O=C(NCC(F)(F)F)[C@](CC)(C)NC1=	O=C(NCC(F)(F)F)[C@](CC)(C)NC1=N

			NC(C2=CNC3=C2C=CC=N3)=NC=C1	C(C2=C(O)NC3=C2C=CC=N3)=NC=C1
39	VU0424238	[64]	CC1=CC(OC2=CN=CN=C2)=CC(C(NC3=CC=C(F)C=N3)=O)=N1	CC1=CC(OC2=CN=CN=C2O)=CC(C(NC3=CC=C(F)C=N3)=O)=N1
40	VU0424238-M1	[64]	CC1=CC(OC2=CN=CN=C2O)=CC(C(NC3=CC=C(F)C=N3)=O)=N1	CC1=CC(OC2=CN=C(O)N=C2O)=CC(C(NC3=CC=C(F)C=N3)=O)=N1
41	PF-05190457	[65]	CC1=CN2C=C(CC(=O)N3CCC4(CN(C4)[C@@H]4CCC5=C4C=CC(=C5)C4=CC(C)=NC=N4)CC3)N=C2S1	CC1=CN2C=C(N=C2S1)CC(N3CCC4(CC3)CN([C@@H]5CCC6=C5C=CC(C7=CC(C)=NC(O)=N7)=C6)C4)=O
42	Cryptolepine	[66]	C[N+]1=C2C(C=CC=C2)=CC3=C1C(C=CC=C4)=C4N3	C[N+]1=C2C(C=CC=C2)=C(O)C3=C1C(C=CC=C4)=C4N3
43	N1-methylnicotinamide	[67]	NC(C1=CC=C[N+](C)=C1)=O	NC(C1=C(O)C=C[N+](C)=C1)=O
44	2-Methylquinazoline	[68]	Cc1nc(c2cn1)cccc2	Cc1nc(O)c2ccccc2n1
45	Quinazoline_Beedham_Q6	[68]	C(/c1cnccc1)=C\c2nc(c3cn2)cccc3	Oc1nc(/C=C/c2cccnc2)nc3ccccc31
46	Quinazoline_Beedham_Q7	[68]	c1cc[n+](Cc2nc(c3cn2)cccc3)cc1	Oc1nc(C[n+]2ccccc2)nc3ccccc31
47	Quinazoline_Beedham_Q8	[68]	OCC(c1c(c2ncn1)cccc2)CO	OCC(CO)c1nc(O)nc2ccccc12
48	Quinazoline_Beedham_Q9	[68]	c1cc(c2cc1)ncnc2N3CCCCC3	Oc1nc(N2CCCCC2)c3ccccc3n1
49	Quinazoline_Beedham_Q10	[68]	O=S1(N(CCC2CCN(c3c(c4ncn3)cccc4)CC2)CCCC1)=O	O=S1(N(CCCC1)CCC2CCN(CC2)c3nc(O)nc4ccccc34)=O
50	Quinazoline_Beedham_Q11	[68]	COc1cc(c2cc1)c(N3CCC(CCN4S(=O)(=O)CCCC4)CC3)ncn2	COc1ccc2nc(O)nc(N3CCC(CC3)CCN4S(=O)(CCCC4)=O)c2c1
51	Quinazoline_Beedham_Q14	[68]	CCNOCOC1CCN(c2c(c3ncn2)cccc3)CC1	CCNOCOC1CCN(CC1)c2nc(O)nc3ccccc23
52	Quinazoline_Beedham_Q13	[68]	COc1c(OC)cc(c2c1)c(N3CCC(CCN4S(=O)(=O)CCCC4)CC3)ncn2	COc1cc2nc(O)nc(N3CCC(CC3)CCN4S(=O)(CCCC4)=O)c2cc1OC
53	1-Cl-67-DiMeO-phthalazine	[68]	Oc1c(O)cc(c2c1)c(Cl)nnc2	Oc1cc2c(O)nnc(Cl)c2cc1O

Table S2. The number of the substrate and the SOM associated with hAOX in the training set and external test set

	Substrate	SOM	Non-SOM
Training set	198	322	315
Test set	53	56	112

Table S3. The parameters and value of feature selection methods

Feature selection method	Parameters
Variance Threshold (VT)	Threshold = 0
Select percentile of Feature (SPF)	Percentile = 5,10,15, 20, 25, ..., 95
Principal Component Analysis (PCA)	n_components = range (1, min(x_sample,x_feature))

Table S4. Parameters of the machine learning methods

Machine learning method	Parameters
GDBT	"n_estimators": range (10, 101, 10), "learning_rate":np.arange(1, 21,1)*0.1
SVM	'kernel': ['rbf'], 'gamma': np.logspace(-15, 3, 10, base=2), 'C': np.logspace(-5, 9, 8, base=2), 'class_weight': ['balanced']
RF	"min_samples_split": range (3, 5),"min_samples_leaf": range (3, 5),"n_estimators": range (10, 121, 10),"criterion": ["gini", "entropy"],"class_weight": ["balanced_subsample", "balanced"]

Table S5. The definition of atom features for the WLN model

Atom feature	Description
Atom type	One hot vector specifying the type of this atom: ['C', 'N', 'O', 'S', 'F', 'Si', 'P', 'Cl', 'Br', 'Mg', 'Na', 'Ca', 'Fe','As', 'Al', 'I', 'B', 'V', 'K', 'Tl', 'Yb', 'Sb', 'Sn', 'Ag', 'Pd', 'Co', 'Se', 'Ti', 'Zn', 'H', 'Li', 'Ge', 'Cu', 'Au', 'Ni', 'Cd', 'In', 'Mn', 'Zr', 'Cr', 'Pt', 'Hg', 'Pb', 'W', 'Ru', 'Nb', 'Re', 'Te', 'Rh', 'Tc', 'Ba', 'Bi', 'Hf', 'Mo', 'U', 'Sm', 'Os', 'Ir', 'Ce', 'Gd', 'Ga', 'Cs']
Charge	Electrostatic charge of this atom: [-3, -2, -1, 0, 1, 2]
Degree	The degree of the atom: range (5)

Table S6. The definition of bond features for the WLN model

Bond feature	Description
Bond type	One hot vector of Single, Double, Triple, Aromatic
Conjugated	whether the bond is conjugated
In rings	whether the bond is in a ring of any size

Table S7. Configuration for the WLN model

Parameters	Value
Batch size	20
Hidden size	300
max_norm	5.0

node in_feats	82
edge_in_feat	6
node_pair_in_feats	10
n_layers	3
Learning rate	0.001

Table S8. Configuration for the Transformer model

Parameters	Value
rnn_size	400
layers	6
transformer_ff	512
heads	8
Optim	adam
adam_beta1	0.9
adam_beta2	0.998
decay_method	noam
learning_rate	2.0
batch_size	500
batch_type	tokens
dropout	0.1
label_smoothing	0.1

Table S9. The predicted results of the transformer-baseline model on the test set

Reactant	C1=CC=C2C=NN=CC2=C1		
Product	OC1=NN=CC2=CC=CC=C21		
Top-k	Predicted SMILES	Validity	Accurate
Top-1	CC1=CC=C2N=CN=C(O)C2=C1	Valid	Wrong
Top-2	CC1=CC=C2N=C(O)N=CC2=C1	Valid	Wrong
Top-3	OC1=CC=C2C=CC=CC2=N1	Valid	Wrong
Top-4	CC1=CC=C2N=CO)N=CC2=C1	Invalid	Wrong
Top-5	CC1=CC=C2N=CN=C(O)C2=	Invalid	Wrong

Table S10. The predicted results of the transformer-transfer learning model on the test set

Reactant	C1=CC=C2C=NN=CC2=C1		
Product	OC1=NN=CC2=CC=CC=C21		
Top-k	Predicted SMILES	Validity	Accurate
Top-1	OC1=C2C=CC=CC2=CN=N1	Valid	Right
Top-2	OC1=NN=CC2=C1C=CC=C2	Valid	Right

Top-3	OC1=NN=CC2=CC=CC=C21	Valid	Right
Top-4	OC1=NN=CC2=CC=CC=C12	Valid	Wrong
Top-5	OC1=C2C=CC=CC2=C(O)N=N1	Valid	Wrong

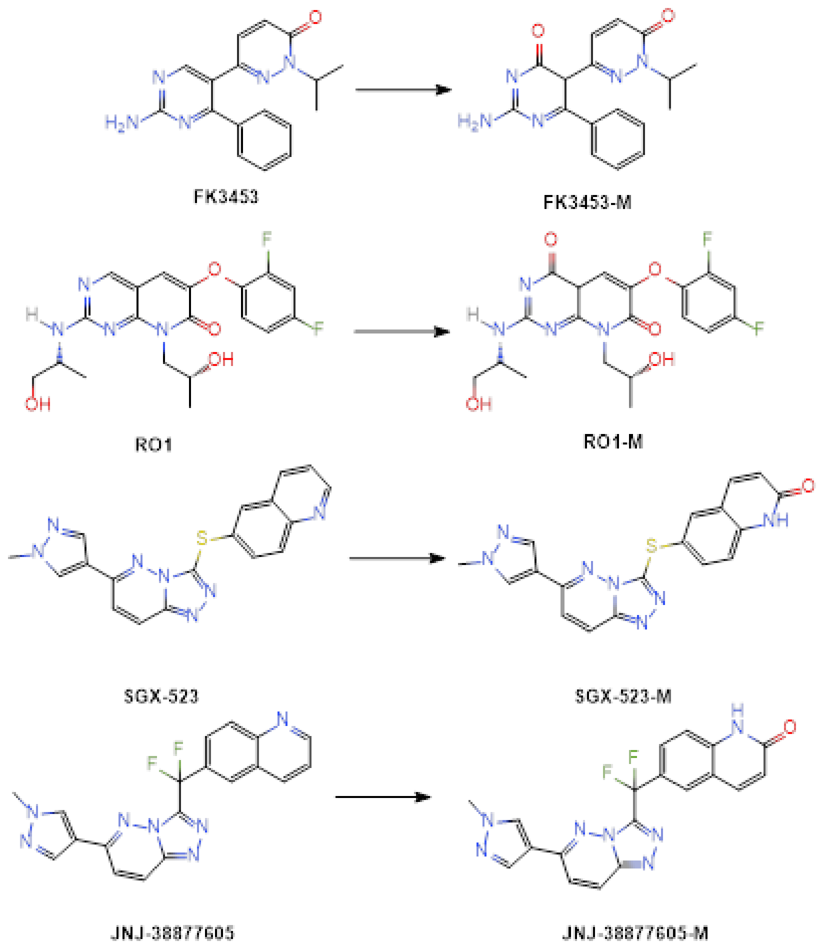

Figure S1. Examples of hAOX -catalyzed reactions.

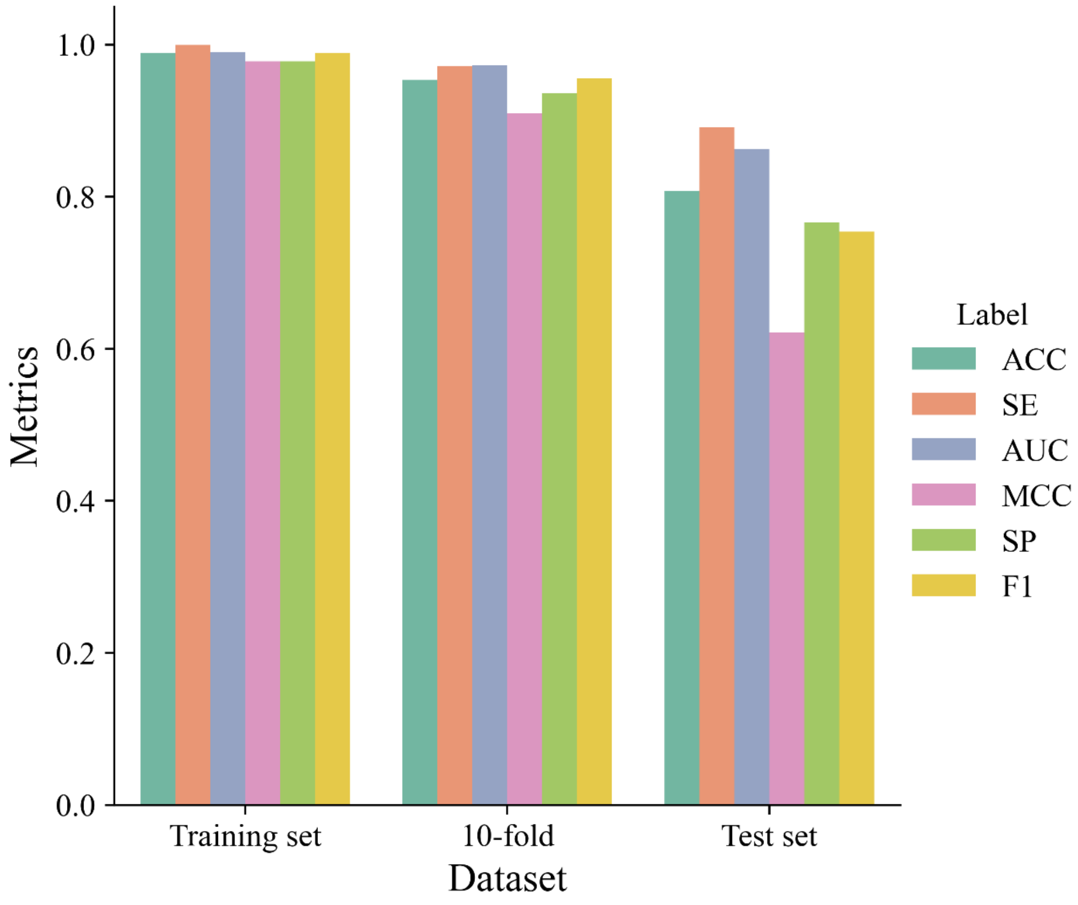

Figure S2. The performance of the best fingerprint-based model on training set, 10-fold and test set.

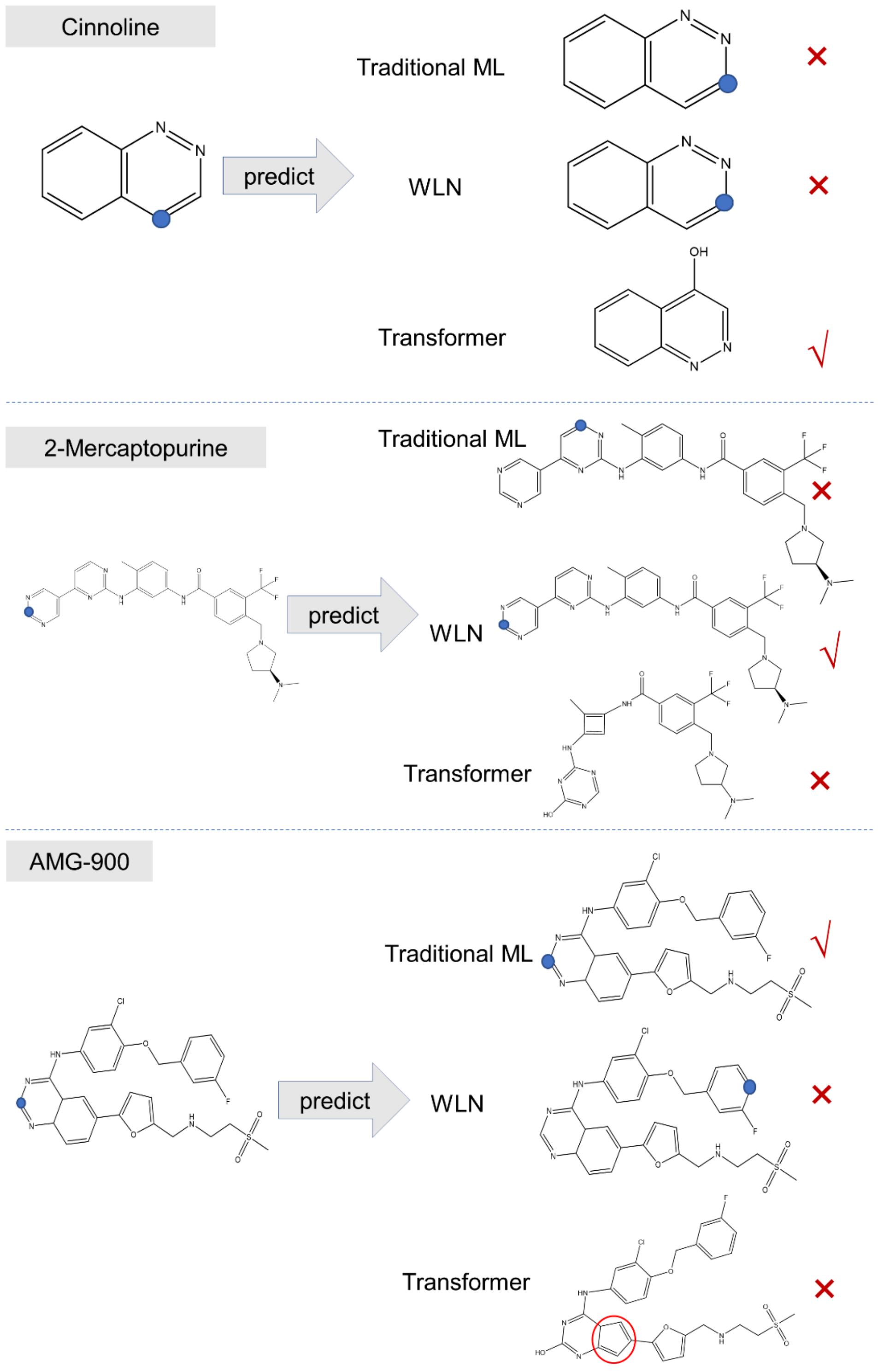

Figure S3. Some examples of results predicted by the three different model.
